# RBM22, a Key Player of Pre-mRNA Splicing and Gene Expression Regulation, Is Altered in Cancer

**DOI:** 10.3390/cancers14030643

**Published:** 2022-01-27

**Authors:** Benoît Soubise, Yan Jiang, Nathalie Douet-Guilbert, Marie-Bérengère Troadec

**Affiliations:** 1 Université de Brest, Inserm, EFS, UMR 1078, GGB, F-29200 Brest, France; benoit.soubise@univ-brest.fr (B.S.); jiangyanjdyy@jlu.edu.cn (Y.J.); nathalie.douet-guilbert@chu-brest.fr (N.D.-G.); 2 Department of Hematology, The First Hospital of Jilin University, Changchun 130021, China; 3 CHRU Brest, Service de Génétique, Laboratoire de Génétique Chromosomique, F-29200 Brest, France

**Keywords:** RBM22, pre-mRNA splicing, alternative splicing, RBP, RNA binding protein, RRM motif, myelodysplasia, gene regulation, cancer

## Abstract

**Simple Summary:**

RBM22 is a gene that encodes an essential RNA-Binding Protein involved in pre-mRNA splicing and transcription, with a DNA-binding function. This dual RNA/DNA-Binding activity provides a new insight of the regulation of gene expression by RBM22. Many studies have reported that RBM22 is essential for cell survival, mitosis, and differentiation processes. Consequently, RBM22 alterations are observed in several diseases and notably in cancer. It is suggested that RBM22 haploinsufficiency and dosage may be critical for its proper function. In this review, we aim at making a state-of-the-art review of this intriguing gene, encoding an RNA/DNA-binding protein that is understudied, and that could represent a potential therapeutic target in specific diseases and cancer.

**Abstract:**

RNA-Binding Proteins (RBP) are very diverse and cover a large number of functions in the cells. This review focuses on RBM22, a gene encoding an RBP and belonging to the RNA-Binding Motif (RBM) family of genes. RBM22 presents a Zinc Finger like and a Zinc Finger domain, an RNA-Recognition Motif (RRM), and a Proline-Rich domain with a general structure suggesting a fusion of two yeast genes during evolution: *Cwc2* and *Ecm2*. RBM22 is mainly involved in pre-mRNA splicing, playing the essential role of maintaining the conformation of the catalytic core of the spliceosome and acting as a bridge between the catalytic core and other essential protein components of the spliceosome. RBM22 is also involved in gene regulation, and is able to bind DNA, acting as a bona fide transcription factor on a large number of target genes. Undoubtedly due to its wide scope in the regulation of gene expression, RBM22 has been associated with several pathologies and, notably, with the aggressiveness of cancer cells and with the phenotype of a myelodysplastic syndrome. Mutations, enforced expression level, and haploinsufficiency of *RBM22* gene are observed in those diseases. RBM22 could represent a potential therapeutic target in specific diseases, and, notably, in cancer.

## 1. Introduction

Proposed to be the first genetic-information carrier for the history of life [1], ribonucleic acids (RNA) are one of the most important molecules—if not the most important—in every living organism. Mostly known as the intermediate between DNA and proteins, it is no longer necessary to prove that RNA is also involved in many other processes. During its whole lifetime, an RNA molecule will be in contact with numerous proteins. All of these RNA-Binding Proteins (RBP) must have at least one RNA-Binding Domain (RBD) to do so [2]. Numerous RBDs are involved in the binding to a large variety of types and secondary structures of RNA. In 2014, Gerstberger and coworkers studied the extent of RBPs and RBDs within the human genome. Among the 1542 RBPs (encoded by 7.5% of all human protein-coding genes) reported in the Protein families (Pfam) database, the team identified ~600 different RBDs [3]. Most of them are found in only 1 or 2 genes and only 20 classes of RBDs are found in more than 10 genes. These domains include: the K homology domain (KH), the Zinc Finger type CCCH, the DEAD motif, the Arginine-Glyine-Glycine (RGG) box, and, most frequently, the RNA-Recognition Motif (RRM) [3].

RNA-Binding Proteins are involved in numerous RNA-related processes. The majority of them are involved in protein synthesis. Most of them interact with mRNA and pre-mRNA and are involved in their processing: splicing, stabilization, and transport [3,4,5]; the rest are ribosomal proteins, or proteins involved in the biogenesis of all the necessary components for protein synthesis: tRNA, snRNA, snRNPs, rRNAs, or snoRNAs [2]. Additionally, a non-negligible proportion of RBPs (~10%) are involved in non-coding RNA (ncRNA) processing, and thus, in all the functions that accompany it [3,6].

It is important to note that many RBPs are able to contact several types of RNA at a time due to an important feature: most of them bear more than one RBD in their structure. It can either be a repetition of the same RBD, or other types of RBDs [7]. This allows the proteins to have more complex roles, such as, for instance, putting pre-mRNA and snRNAs in close proximity during pre-mRNA splicing.

RBM22 is a gene encoding an RBP that is mostly involved in pre-mRNA splicing, which has been, for a long time, understudied. However, recent studies have revealed the major role that RBM22 plays in pre-mRNA splicing and have shown its implication in different malignancies, thus arousing new interest for this gene.

In this review, we aim at making a state-of-the-art review of the current knowledge about RBM22, from its normal function to its pathological role.

## 2. The RNA-Binding Motif (RBM) Family

### 2.1. The Unity of the Family: The RNA-Recognition Motif

The RNA-Binding Motif (RBM) family was created two decades ago to fulfil the need of classifying a group of genes, of which their function was then not really known, on the basis of a common structural feature: the presence of at least one RNA-Recognition Motif (RRM) in their sequence [8]. Among all the RNA-Binding Domains (RBD), the RRM is the most frequent in higher vertebrates, being present in 10% of all human RBPs, and representing 0.5 to 1% of all human genes [3]. The RRM is found in many other proteins such as hnRNPs, nucleolin, and poly(A)-binding proteins, which are involved in RNA processing [9].

The RRM, which is about 90 amino acids long, presents a relatively conserved structure of four antiparallel β-strands, forming a β-sheet, packed against two α-helices, following the sequence β_1_α_1_β_2_β_3_α_2_β_4_ [10]_._ Most commonly, the RRM binds single stranded RNA by three aromatic residues located in 2 conserved subdomains: RNP1 and RNP2, respectively, found in β_3_ and β_1_ strands. The interaction with RNA is made by the bond between a dinucleotide and two aromatic cycles in RNP1 and RNP2. A third aromatic cycle, also located in RNP1, gets in between the two sugars of the dinucleotide [10]. More precisely, the 5′ nucleotide binds to the RNP2 aromatic cycle and the 3′ nucleotide binds to the RNP1 C-terminal aromatic cycle. However, the RRM is well known for its very high variety of conformations, allowing it to interact with different types and shapes of RNA, or even to interact with proteins [10,11]. In most cases, additional elements in the external parts of the RRM are able to modulate the sequence specificity and affinity of the bond. The presence of additional β-strands or α-helices can change the capacity of the domain to bind RNA. Some elements will increase the specificity and/or the affinity (e.g., by increasing the surface available for the interaction or, sometimes, changing the conformation of the domain), while other elements will lower it [12]. The RNA-binding capacity can also be increased by the internal loops of the RRM, which can contact the RNA [13], or by the presence of β-hairpins, which allow protein-protein interactions [14,15,16]. Alternatively, some proteins present multiple RBDs, eventually including RRMs, modifying the RNA-binding capacity of the protein [17]. At last, one feature of the RRM that is important to highlight is its capacity of accommodating H_2_O molecules in order to modulate its RNA sequence specificity [18,19]. This allows it to be less specific to an RNA motif.

Thereby, due to this variability in RRM structures and functions, a single RRM is able to contact up to 8 ribonucleotides in a single-stranded manner at a time [20,21,22,23,24]. Additionally, due to those differences in RRMs, an up-to-date, no consensus RNA sequence is described for this domain. Nevertheless, some RRMs show sequence specificity over their binding with RNA, such as RBM20 RRM, whose structure has recently been resolved [25]. However, defining a consensus sequence for all the RRMs seems yet unfeasible.

In an attempt to reference and organize the data on RRMs, Nowacka and colleagues developed a database compiling information about all the existing RRMs: RRMdb (available online: http://iimcb.genesilico.pl/rrm/) (accessed on 26 November 2021). Briefly, starting with a core of well-characterized RRM motifs from SCOP and PFAM databases, they interrogated the NCBI protein database for additional RRM-like motifs using PSI-BLAST. The obtained sequences were filtered with CD-HIT and clustered into 415 families using different Markov algorithms. Next, the families were associated with the relevant literature and organized into a network according to their pairwise sequence similarity. This tool identifies 415 families of distinct RRMs [26]. However, some clusters of RRM, determined by sequence homology, show evidence of a certain homogeneity between different families. Forty-six families of RRM are found in *RBM* genes (Table 1). Of note, *RBM22* is the sole member of its RRM family (number 77). Although, its family is identified as closely related to the family of its yeast homologue *Cwc2* (number 142).

### 2.2. The Functions of RBM Proteins in RNA-Related Metabolism

RBM proteins, consisting in a family of about 50 members, are involved in RNA metabolism due to their capacity of binding this molecule (Table 2). Most RBMs are directly involved in multiple steps of pre-mRNA splicing. For instance, RBM5 and RBM17 are implicated at the very beginning of splicing where the intron and its junctions with the exons are recognized by the first components of the spliceosome (A complex) [27,28], while RBM22 intervenes during the first trans-esterification of splicing [29] and RBM8 is involved in the late stages of the process [30]. Many RBM proteins are regulators of the splicing process, such as RBMY1, RBM9, RBM10, and RBM24, which regulate alternative splicing [31,32,33,34]. Their functions also comprise RNA metabolism-related processes, such as RNA-transport, stability, or translation. For instance, RBM26/27, by its interaction with the Poly(A) tail eXosome Targeting (PAXT) Complex, modulates the degradation of some RNAs [35]. RBM3, which is mainly located in the cytoplasm, modulates the translation of some mRNAs in response to a cold-shock [36]. All the functions of the *RBM* family members are compiled in Table 2, based on data found in the literature and databases (Genecards^®^, Uniprot, Ensembl).

Importantly, due to their activity with RNAs, RBM members are involved in crucial biological functions like apoptosis, control of the cell cycle, and the differentiation of several cell types. This inevitably leads to a large number of diseases when their function is altered. Recently, Li and coworkers reviewed the role played by RBM members in cancer [140].

In this review, we particularly focus on the cellular and molecular functions of *RBM22*, due to its crucial role in pre-mRNA splicing.

## 3. RBM22 Is an RNA-Binding Protein

### 3.1. Structural Features of RBM22

**Figure 1:**
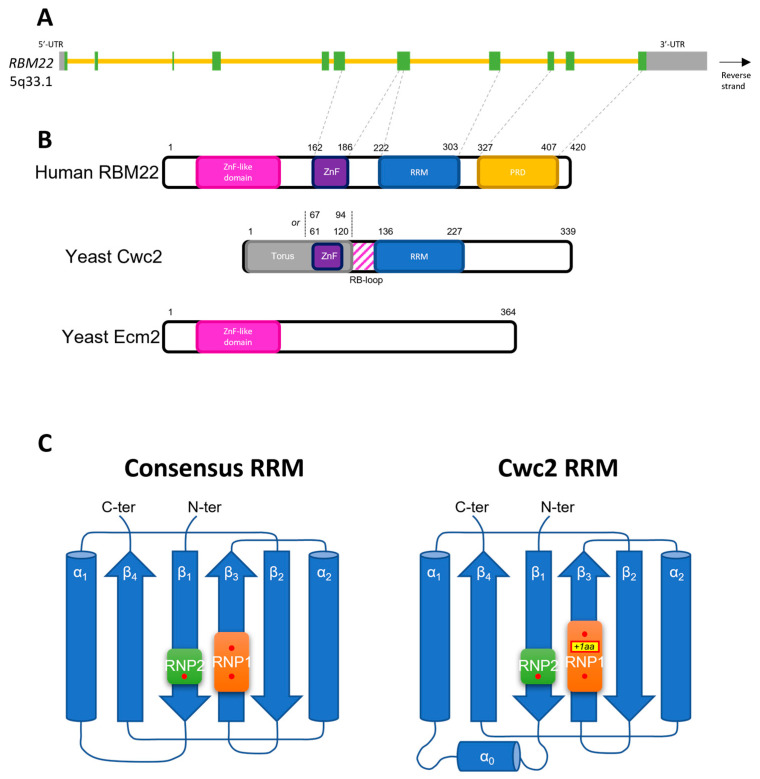
(**A**) Scheme of *RBM22* gene. Exons are represented by green and grey boxes when they are translated and are either in the 5′-UTR or 3′-UTR, respectively. Introns are represented by yellow lines. The correspondence of exons with the domains of the protein is indicated, when known, with the grey dashed line between (**A**) and (**B**); (**B**) Scheme of the domains of the human protein RBM22 and the two yeast proteins Cwc2 and Ecm2. The structural homology between the three proteins suggests that *RBM22* is a fusion gene of the two yeast genes; (**C**) Two-dimension representation of the structure of the RNA-Recognition Motif (RRM). Left panel shows the consensus structure of the RRM. Right panel shows the RRM of Cwc2, the yeast orthologue of RBM22. The latter presents an additional amino acid between the two aromatic residues of RNP1, and another α-helix between β_1_ and α_1_. The aromatic residues of the RNP subdomains are represented by red dots. This 2D representation aims at respecting as much as possible the adjacent structures, as described in [10]. For this representation, the structures are annotated in a N-ter to C-ter manner, from 1 to 4. The α-helix 1 in the right panel corresponds to α-helix 9 in [141]. ZnF: Zinc Finger Domain; RRM: RNA-Recognition Motif; PRD: Proline-Rich Domain; Aa: Amino acid.

*RBM22*—also referred to as *ZC3H16*, *FSAP47,* or *FLJ10290*—is a 10,318 base-pair gene located on the reverse strand of the long arm of chromosome 5, at the locus 5q33.1 in human. Containing 11 exons, this gene encodes a protein of 420 amino acids, with an apparent molecular weight of 46.9 kDa (Figure 1A,B). Other transcripts are described, but none of them are predicted as protein-coding.

In its N-terminal region, RBM22 contains a Zinc Finger like domain and a CCCH Zing-Finger Domain of the uncommon type C_7_C_5_C_3_H from amino acids 162 to 186, which is described as an extended loop able to bind a zinc ion [29,97,141,142]. Additionally, in accordance with the common feature of the other *RBM* members, the protein RBM22 contains an RNA-Recognition Motif (RRM), which is located in its C-terminal portion, from amino acids 222 to 303 [29] (Figure 1B). Finally, it also contains a Proline-Rich Domain (PRD) in its C-terminal region, the function of which is not yet described and is broadly unclear in RBPs. Only a few studies described in the past a role in RNA/DNA-binding or protein-protein interactions [143,144,145]. The arrangement of these domains shows a strong evidence of a fusion of two yeast genes: *Ecm2* and *Cwc2* (Figure 1B) [97]. Indeed, RBM22 carries structural homology with Ecm2 in its N-terminal region containing the Zinc Finger like domain, and structural homology with Cwc2 in its C-terminal region, which contains the Zinc Finger (ZnF) Domain and the RRM. However, the region downstream of the RRM, containing the PRD, is less conserved between the yeast Cwc2 and RBM22 [142].

Though RBM22′s RRM is not extensively described, its function and structure can be predicted by the homology it shares with Cwc2′s RRM. The latter is structured with the common sequence β_1_α_1_β_2_β_3_α_2_β_4_ and the two domains RNP1 and RNP2 are located, respectively, in β_3_ and β_1_. Nevertheless, Cwc2 RRM shows some atypical features such as a small additional α-helix between β_1_ and α_1_ (Figure 1C), and an additional residue between the two aromatic residues in RNP1 [142]. Surprisingly, the other domains of Cwc2 are required for its RRM to bind RNA [141], maintaining it in a tight conformation with its neighbor ZnF Domain with the help of the Torus domain and a small loop between them [146]. This loop is called the RNA-Binding loop (RB-loop); the positively charged amino acids in its C-terminal portion facilitate RNA binding [141]. Nevertheless, in contrast with the aromatic residues of the RRM, which are highly conserved between yeast and human, the RB-loop residues are poorly conserved and there seems to be more residues in the human RBM22 RB-loop [29,141].

### 3.2. RBM22 Is Essential for Gene Expression through Transcription Regulation and Pre-mRNA Splicing

#### 3.2.1. RBM22 Stabilizes the Catalytic Core of the Spliceosome

Precursor messenger RNA (Pre-mRNA) splicing is the process by which an immature RNA—containing introns and exons—is rearranged to produce a mature messenger RNA (mRNA). It is a very complex process, and thus, it will only be briefly described here.

**Figure 2:**
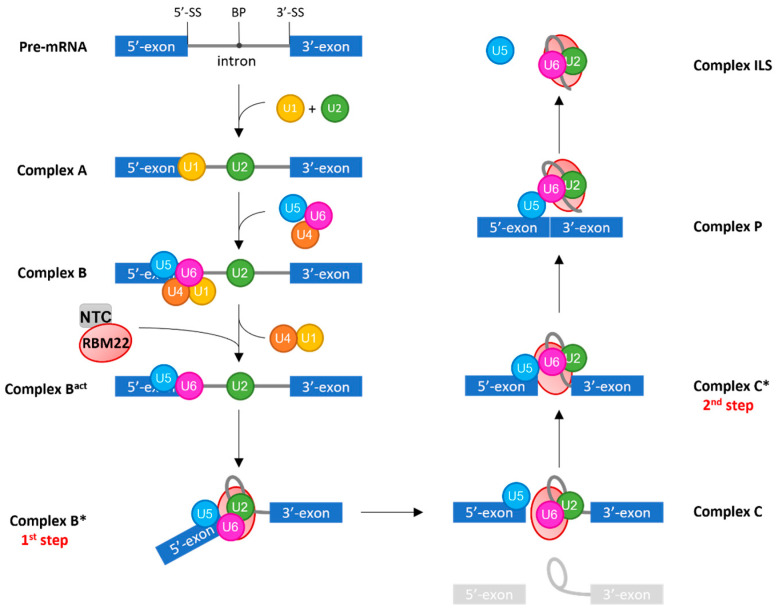
Brief scheme of pre-mRNA splicing. U1 and U2-snRNAs first recognize specific sequences: U1 base-pairs with the 5′-splice site (5′-SS) and U2 base-pairs with the Branch Point (BP) sequence, forming Complex A. The preformed tri-snRNP U4/U6.U5 then arrives on the complex, near the 5′-SS, to form the B Complex. In the B^act^ Complex, U5-snRNA binds exonic sequences while U6-snRNA takes the place of U1; thus, the latter leaves the complex accompanied by U4-snRNP. It is also in this complex that the NTC and RBM22 arrive. To form the B* complex, U6-snRNA will then undergo conformational changes, forming its Internal Stem-Loop and a helix with U2-snRNA. This conformation, maintained by RBM22, corresponds to the catalytic core of the spliceosome. In the B* complex, the first transesterification reaction, corresponding to the first step of splicing and also called branching, reaction occurs. It produces an intronic semi-lariat intermediate and a free 5′-exon corresponding to Complex C. The second transesterification reaction, corresponding to the second step, occurs in the C* complex. It ligates the two exons to form a mature mRNA. The ligated exons are then removed from the Post-catalytic complex (P), leaving the rest of the spliceosome with the intron lariat (ILS Complex). The position of RBM22 after the C* Complex is unclear, and thus is not represented. At each step of the process, the different complexes are subjected to crucial conformation changes carried out by ATPase proteins. 3′-SS stands for 3′ splice site.

Occurring simultaneously with transcription in the nucleus [147], pre-mRNA splicing is carried out by the spliceosome, a complex involving small nuclear ribonucleoproteins (snRNPs) associated with their respective small nuclear RNAs (snRNAs), called U1, U2, U4, U5, and U6 snRNP/snRNA, and over 100 associated proteins [148]. Several conserved sequences of the pre-mRNA will first be recognized by the snRNAs: U1-snRNA binds the splice site located in the 5′-end of the intron (5′-SS) and U2-snRNA binds the Branch Point (BP) located in the second half of the intron (Figure 2) [149,150]. Then the preformed tri-snRNP U4/U6.U5 arrives on the complex: U5 binds exonic sequences such as a sequence right upstream of the intronic 5′-SS, while U6-snRNA binds a sequence downstream of the 5′-SS by its ACAGA box, destabilizing its interaction with U4-snRNA and taking the place of U1-snRNA. Thus, U1 and U4-snRNAs leave the complex. This change in base-pairing allows U6-snRNA to form a helix with U2-snRNA, encompassing its AGC triad, and an additional internal stem-loop (hereafter, U6-ISL). The ACAGA box, the triad, and the U6-ISL are the 3 essential components of the spliceosome catalytic core. Two metal ions will be bound by the catalytic core: one by the bulge of the U6-ISL, and the other one by the triad of U6/U2 helix Ib. Those ions are then used to catalyze the splicing reactions [151].

Simultaneously, the structure and conformation of the complex is sequentially remodeled by DExD/H-box ATPases/Helicases (and successively named complex A, B, B^act^, B*, C, C*, P, and ILS) with highly dynamic protein interactions. During these conformation changes, two transesterification steps will occur in order to remove the intron. The first transesterification step, called branching reaction, occurs in the catalytically activated B* complex and leads to the formation of an intron semi-lariat due to a nucleophilic attack from the 2′-hydroxyl of the adenosine in the BP on the guanosine of the 5′-SS [98,152,153]. Thus, in the C complex, the 3′-end of the 5′-exon is “free,” but yet, it is maintained in the spliceosome. In the transition C-to-C*, the complex gets catalytically activated for the second transesterification step, the exon ligation, in which another attack from the 3′-hydroxyl of the 5′-exon to the phosphate of the 3′-SS will occur. Thereby, in the post-catalytic complex (P), the two exons are ligated. They will then be removed from the complex, leaving the intron-lariat with the rest of the components; this is the Intron-Lariat Spliceosome Complex (ILS). Finally, all the components will be recycled for another splicing reaction on the same pre-mRNA or another one. The entire process is only possible thanks to the specific conformation adopted by the catalytic core of the spliceosome, at each step, which is governed by all its protein partners.

**Figure 3:**
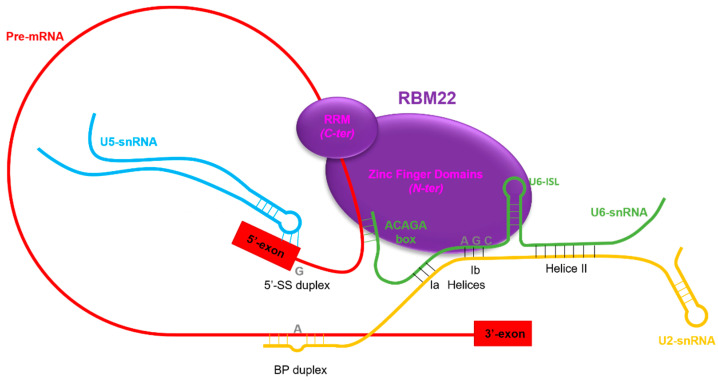
Location of RBM22 in the B^act^ complex of the spliceosome. RBM22 contains a Zinc Finger like domain, a Zinc Finger domain and an RNA-Recognition Motif. Through these three domains, it is able to contact several parts of the catalytic core of the spliceosome: the Internal Stem Loop of U6 snRNA (U6-ISL); the ACAGA box of U6 snRNA, which interacts with the 5′-splice site (5′-SS) of the pre-mRNA; and it also contacts and sequesters the pre-mRNA downstream of the 5′-SS. By all those contacts, RBM22 maintains an active conformation of the catalytic core of the spliceosome, and thus, it plays an essential role in the splicing process.

RBM22 is involved in maintaining the proper conformation of the catalytic core of the spliceosome. It is considered a NineTeen Related (NTR) protein, which arrives on the spliceosome with the rest of the Nineteen Complex (NTC) during the remodeling B-to-B^act^ by Brr2 (Figure 2) [149,154]. RBM22 is able to contact several components of the spliceosomal catalytic core, in accordance with its homology with the two yeast proteins Cwc2 and Ecm2 (Figure 3). By its N-terminal region, containing the ZnF-like domain homologous to that of Ecm2 and the ZnF domain homologous to that of Cwc2, RBM22 is in direct contact with a few nucleotides of U6-snRNA, downstream of the U6-ACAGA box, and a few nucleotides of the pre-mRNA, downstream of the 5′-SS [97]. Recent studies described crystal structures in which a part of the N-terminal region of RBM22 is folded in a positively charged channel where the pre-mRNA is locked down [29,155]. The presence of positively charged residues thus allows a direct protein-RNA interaction. However, it is still unclear whether RBM22 is partially unfolded when arriving in the complex to bind the pre-mRNA or if it needs to bind the pre-mRNA to correctly fold up [29]. A very elegant hypothesis is that the RRM of RBM22 would transiently contact the U2 helix IIb, maintaining it in an opened conformation. This would allow it contacting the intron before disruption of the interaction with U2 and wrapping around the intron [156]. Surprisingly though, while the RRM of the yeast Cwc2 is included in a single folding unit located over the region of the U6-ISL and the pre-mRNA, downstream of the 5′SS [141], it is excluded from that region in the human spliceosome, and is located in a more downstream region of the intron [29,98,153]. Thus, by its RRM, RBM22 binds a few nucleotides downstream of the U6-ACAGA box and interacts with the β-barrel of the RNA helicase Aquarius, maintaining the rest of the intron away from the catalytic center of the spliceosome and potentially preventing it from interfering with the reaction [29,155].

Nevertheless, RBM22 holds the pre-mRNA from its arrival, in the B^act^ complex, until the release of the intron lariat, in the ILS complex [29,97,98,99,100,101]. Additionally, RBM22 also contacts the U6-ISL. Interestingly, the bound between RBM22 and the U6-ISL is relatively weak in the B^act^ complex and becomes stronger during the first step of splicing [97]. This suggests a change of conformation of the U6-ISL during the first step of splicing, leading to a stronger binding by RBM22 [97,157]. Thus, by its N-terminal region and its contacts with several elements of the catalytic core, RBM22 plays the role of an “RNA-folder,” maintaining an active conformation during the first transesterification step of splicing. Indeed, several experiments showed that the absence of the yeast orthologue Cwc2 was critical for the catalytic core and the first step of splicing; a solution structure of the yeast U2/U6-snRNA complex showed that the three elements of the catalytic core were away from each other, in an unfolded conformation [157]. Likewise, an in vitro splicing experiment showed that spliceosomes lacking Cwc2 were unable to achieve the first step of splicing. The correct splicing could be restored when the spliceosomes were complemented with recombinant Cwc2 [97].

Regarding the second step of splicing, RBM22 has been found in the C and C* complexes, as mentioned earlier. However, up-to-date, its role in these complexes has not been deeply studied.

Finally, it is interesting to note the reflection of Hoskins’ team, which states that RBM22 more accurately mimics Ecm2 instead of Cwc2, in terms of interaction with the U6-snRNA. Thus, they believe Ecm2 is a closer structural homolog of RBM22 [156]. However, the evolution of RBM22 has never been truly studied. Therefore, even if the literature mainly underlines the analogies between RBM22 and Cwc2, making it appear, sometimes, as its main homolog and forgetting about Ecm2, their evolution must be clarified to better understand the origin and the role of each domain of RBM22.

#### 3.2.2. RBM22 Organizes Several Protein Components around the Catalytic Core

Aside from its “RNA-folder” role, RBM22 is also important for the architectural organization of the spliceosome. Indeed, RBM22 belongs to the NineTeen Related Complex (NTR Complex), a group of 18 proteins associated to the NineTeen Complex (NTC), which itself is a group of 8 proteins associated to the central PRP19 protein [152]. However, what is often called the NTR does not seem to be a real protein complex, but a set of proteins that independently come and go with the NTC [154].

The NTC is an essential, canonical component of the spliceosome, conserved from yeast to human [154]. It arrives on the spliceosome during the B-to-B^act^ remodeling, along with RBM22, before U4-snRNA removal. The NTC plays a major role in specifying the interactions between the snRNAs and the pre-mRNA, ensuring the fidelity of splicing, but also in maintaining and stabilizing an active conformation of the catalytic core of the spliceosome during both steps of splicing [154]. Indeed, Cheng’s team showed that the absence of the NTC destabilizes the association of U5 and U6-snRNAs with the pre-mRNA [158,159]. Paradoxically, the main core of the NTC is located away from the catalytic core of the spliceosome. Only two proteins make the link between the two regions: RBM22 and SRm300 (respectively, Cwc2 and Cwc21 in *S. cerevisiae*), with RBM22 being the only RNA-binding protein in the NTR in direct contact with the spliceosomal catalytic core [160].

Apart from linking the NTC to the catalytic core of the spliceosome by its interaction with PRP19, RBM22 also interacts with several NTR and non-NTR proteins, which also play a major role in splicing (Table 3). Most of these interactions were established by recent crystallography experiments, and some proteins could be crosslinked to RBM22: Aquarius, Cdc5, Isy1, Prp17, Prp8, and SKIP [153].

#### 3.2.3. RBM22 Depletion Impacts Pre-mRNA Splicing

The apparent major role that RBM22/Cwc2 plays in splicing has been supported by a few depletion experiments. In 2009, McGrail and coworkers showed in the yeast *S. cerevisiae* that Cwc2 was essential for splicing in vivo as its depletion using a Gal-dependent-Cwc2-expression strain led to the accumulation of unspliced transcripts. Of note, they observed an intron retention in the RNA of U3 [142]. Later on, Rasche and coworkers showed that yeast Cwc2-depleted spliceosome extracts failed to achieve the first step of splicing (formation of the intron semi-lariat), leading to an intron retention in the actin RNA. However, Cwc2 was not needed for Prp2 to catalytically activate the complex (remodeling B^act^-to-B*), indicating that the presence of Cwc2 is critical only during the transesterification step. Similarly, they performed in vitro splicing experiments from HeLa RBM22-depleted nuclear extracts. The same observation was made: the absence of RBM22 led to inhibition of the first step of splicing on the synthetic MINX pre-mRNA. In both models (yeast and HeLa extracts), the RNA splicing could be rescued when the depleted extracts were supplemented with Cwc2 or RBM22 [97]. In another study, RBM22 was identified as an alternative-splicing factor in an RNA interference screening in the *Drosophila* S2 cell line. Its knock-down led to the alternative splicing of the exon 4 cluster—containing 12 mutually exclusive exons—of *Dscam* [162].

**Figure 4:**
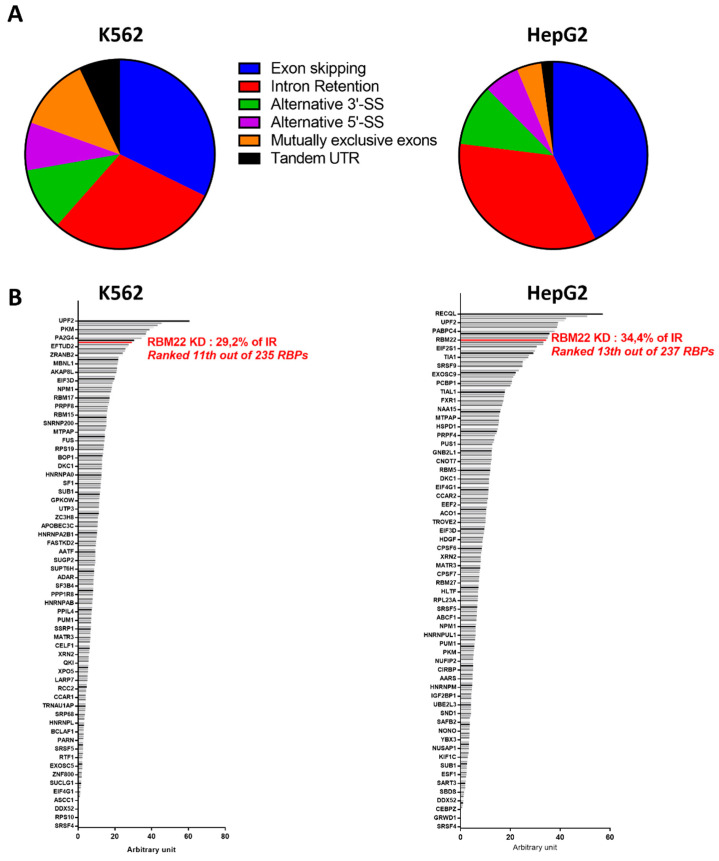
Effect of RBM22 knock-down on pre-mRNA splicing, calculated from data of Van Nostrand et al. [104]. (**A**) Proportion of alternative splicing events due to RBM22 knock-down in K562 and HepG2 cells; (**B**) Proportion of Intron Retention (IR) due to the successive knock-down (KD) of 235 or 237 RNA-Binding Proteins (RBP) in K562 or HepG2 cells, respectively.

Finally, more recently, a very exhaustive study presented a systematic characterization of no less than 356 RBPs in HepG2 and K562 cell lines [104]. The knock-down of RBM22 by small-hairpin RNAs (shRNAs) mainly induced Exon Skipping (ES) and Intron Retention (IR) (Figure 4A). It is interesting to note that the knock-down of RBM22 induced one of the highest proportions of Intron Retention among the RBPs tested: ranked 13th/237 RBPs in HepG2 and 11th/235 RBPs in K562 (Figure 4B) (ranks calculated from data of [104]).

Altogether, those results reaffirm the paramount role that RBM22/Cwc2 plays in the spliceosome, interestingly highlighting its potential implication in alternative splicing, and, notably, intron retentions, when deleted.

#### 3.2.4. Emerging Evidence of Gene Regulation by RBM22

It is well known that many DNA-Binding proteins, notably transcription factors (TF), can also bind RNA. For instance, the master regulator CTCF interacts with several RNAs to be specifically recruited to its target loci [163]. It is also the case for the regulator FUBP1 that is able to bind single-stranded DNA and to activate promoters and enhancers as well as binding RNA [164,165]. Similarly, the TF YY1 was shown to interact with nascent RNA close to its targeted regulatory elements to improve its binding to the chromatin, thus reinforcing and stabilizing the expression of the gene [166].

On this basis, and knowing that transcription co-occurs with RNA processing, as mentioned earlier, a new idea that RBPs could also potentially contact DNA has recently emerged. In 2019, Xiao and coworkers addressed the question leading a large-scale study on 58 and 45 RBPs in HepG2 and K562 cells. They confirmed the aforementioned hypothesis, finding that a large number of RBPs would eventually bind chromatin, mainly around promoters. Moreover, they showed that this binding is enhanced in the presence of the nascent RNA. Interestingly, RBM22 is one of those DNA-binding RBPs, presenting nearly 10,000 ChIP-Seq peaks on the genome, mainly on transcribed regions, with an enrichment on promoters of small RNA genes (tRNA, snoRNA, and miRNA) compared to promoters of lncRNA and protein coding genes. Furthermore, they identified a positive correlation between the expression of the target genes and the probability of RBM22 binding to it. They showed that its binding to the chromatin requires the presence of a nascent RNA as blocking transcription significantly reduced the number of ChIP-Seq peaks on a given region. Strikingly, they observed a deregulation of about 3000 genes when RBM22 was knocked-down. Genes giving rise to an RBM22-bound RNA were significantly more deregulated than those giving non-RBM22-bound RNAs, showing that RBM22 plays a role in transcription and gene regulation, and exhibiting, to some extent, transcription factor-like function. [105]. A coupling between promoter binding and RBP-dependent splicing was not observed in this work but was not definitively excluded either.

Later, Van Nostrand and coworkers confirmed that RBM22 could bind chromatin, mainly on transcribed regions and promoters. They also showed a concordance between eCLIP signals—corresponding to RNAs bound by RBM22—and ChIP-Seq signals, reaffirming the concept of RBP target genes. Finally, in RBM22 knock-down conditions, they also showed a deregulation of a large number of genes (>5000 in HepG2 and >2000 in K562 cells), ranking RBM22 as one of the RBPs that induces the most deregulation. These deregulations were mainly down-regulations (accounting for 60% to 75% of deregulated genes) [104]. This is in contrast with the previous study [105], which showed an equal upward and downward deregulation. Thus, these data need to be strengthened.

These studies provide a new perspective on RBPs, and notably on RBM22, showing that apart from their role in RNA splicing and processing, RBPs are also involved in transcription and gene regulation. This opens a new door in the field of gene expression.

### 3.3. Implication of RBM22 in Ca^2+^-Dependent Regulation of mRNA Splicing

It is well known that calcium plays a major role in signal transduction in the cells. This is in part due to its effect on gene transcription, notably through a few Ca^2+^-dependent transcription factors (e.g., CREB and SRF) [167,168]. It also plays a role in alternative splicing [169].

In 2006, Montaville et al. identified RBM22 as a protein interactor of the calcium-binding protein Apoptosis Linked Gene 2 (ALG-2) by a yeast two-hybrid assay and confirmed their interaction using recombinant fluorescent proteins. Moreover, they showed that overexpressed RBM22 was able to translocate ALG-2 from the cytoplasm to the nucleus [170,171]. In another study, Kreb’s team also showed that, in stress conditions (especially calcium-stress conditions under treatment with Thapsigargin), a higher expression of *RBM22* would enhance the translocation of the second step splicing factor SLU7 from the nucleus to the cytoplasm. Furthermore, the splicing of *Xbp1* was affected. The latter is a transcription factor that is normally alternatively spliced, in endoplasmic reticulum (ER) stress conditions, into the Xbp1-s isoform, which promotes the transcription of ER chaperones. Under treatment with Thapsigargin, the proportion of *Xbp1-s* was greatly reduced when the cells were transfected with vectors expressing *RBM22* and/or *ALG-2* [171]. This highlights a potential role of RBM22 in the Ca^2+^-dependent regulation of splicing that needs to be further studied.

## 4. The Role of RBM22 in Mitosis and Differentiation

### 4.1. Spatio-Temporal Expression of RBM22

According to databases, *RBM22* is a highly conserved gene. It is found in all vertebrates and invertebrates, in plants, and even in yeasts as 2 separated genes, as mentioned above (i.e., *Cwc2* and *Ecm2/Slt11*).

In humans, the expression of *RBM22* is ubiquitous, but it is interestingly more expressed in the bone marrow, where hematopoiesis takes place [172]. Unfortunately, there is no report about the expression of *RBM22* during human development. However, He and coworkers reported its expression during zebrafish development. *zRBM22* was expressed as early as the 1-cell stage, and was then ubiquitously expressed in the fish embryo [173].

### 4.2. Experimental Depletion of RBM22 Results in Mitotic and Differentiation Defects

The effect of *RBM22* depletion on the phenotype of cells and organisms has been reported by several studies. In a Cwc2-depleted yeast model, complementation with versions of Cwc2 carrying mutations on its RRM or ZnF domains induced a growth defect for some mutations [142]. Moreover, in screenings employing RNA interference technologies to deplete a set of genes or every gene one by one, *RBM22* was repeatedly identified as an important or essential gene. In particular, *Rbm22* depletion by RNA interference caused an abnormal development of *Drosophila* embryonic heart [106]. It was also identified as an essential gene for early zebrafish development by insertional mutagenesis [90]. In concordance with this latter result, another *Rbm22* knock-down experiment led to abnormal development of zebrafish head and tail during embryogenesis [173]. However, no link has been established between those phenotypes and any RBM22-defective splicing so far. Recently, using CRISPR/Cas9 technology, Yamauchi et al. identified *Rbm22* as an important, if not essential, gene for the survival of transformed murine cell lines [174]. In 2004, Kittler et al. showed that the knock-down of *RBM22* (called *FLJ10290* at the time) leads to cytokinesis defect and mitotic arrest in HeLa cells, without inducing cell death [102]. As mentioned earlier, *RBM22* is particularly expressed in the bone marrow. Thus, unsurprisingly, Ebert and coworkers showed that *RBM22* haploinsufficiency also impairs the erythroid differentiation of human CD34+ hematopoietic stem or progenitor cells (HSPCs), without impacting megakaryocytic differentiation. This mimicked the phenotype of myelodysplastic syndromes (MDS) [103].

These results and the apparent essentialness of *RBM22* for cell survival and differentiation, as well as organism development, led us to wonder whether *RBM22* could be implicated in human diseases.

### 4.3. Implication of RBM22 in Human Diseases and Cancer: State-of-the-Art and Perspectives

During the last decade, *RBM22* has been reported in a few studies about different pathologies. Of note, if it is shown to be associated to the phenotype of the disease, *RBM22* has never been identified as a driver gene. Herein, we draw on the state-of-the-art research of what is known about *RBM22* in diseases.

According to the COSMIC database (available at cancer.sanger.ac.uk) (accessed on 14 October 2021), *RBM22* is most likely a stable gene, which is rarely mutated [175], reinforcing the idea of its essentialness. Indeed, only a few mutations were reported for *RBM22*—mainly missense and synonymous mutations—with no mutation hotspot. Only two deletions were reported, both in the PRD domain and of 3 nucleotides, leaving the open reading frame unchanged. No fusion gene but a few Copy Number Variants (CNV) were described. This indicates that the pathological role of *RBM22* is rather due to a change in its expression than an altered or modified function. For instance, it was found deregulated in heart tissues of cardio-insufficient patients and overexpressed in non-alcoholic fatty liver patients [132,176]. In the latter case, the knock-down of *RBM22* by RNA interference diminished the expression of lipogenesis enzymes and reduced lipid accumulation in HepG2 cells. Moreover, the authors described two treatments that could influence *RBM22* expression: leptin reduced it while palmitic acid increased it [132].

*RBM22* has also been reported in a few cancers. The OGEE v3 database describes *RBM22* as essential for cancer cells in several human tissues [177]. In 2017, Chan and coworkers reported that *RBM22* is overexpressed in triple-negative breast cancer cells. Moreover, its knock-down reduced the viability of cells [178]. *RBM22* was also found overexpressed in glioblastoma. In that case, the knock-down of *RBM22* affected several functional parameters of the cells: it reduced their proliferation as well as their migration potential; it reduced the secretion of the vascular endothelial growth factor (VEGF) and the number of stem/progenitor cells in the tumorsphere; and finally, it increased apoptosis [179]. Surprisingly however, *RBM22* was recently found to promote the proliferation of gastrointestinal cancer cell lines without any change in its expression. Nevertheless, its knock-down still strongly inhibited the proliferation of cells. Interestingly, only cancer cells were affected by the down-expression of *RBM22* as non-cancer cells did not show any significant reduction of proliferation. Thereby, for the first time, *RBM22* was proposed as a therapeutic target [180]. At the opposite of the aforementioned data, the overexpression of *RBM22* in lung cancer improved the prognosis of the patients [181]. All these data suggest a critical dosage for RBM22 normal function.

Finally, in accordance with the higher expression of *RBM22* in hematopoietic tissues mentioned earlier, *RBM22* alterations are also reported in hematological malignancies. In 2007, *RBM22* was identified as one of the most downregulated genes in the 5q-syndrome, a subtype of myelodysplastic syndrome [182]. The 5q- syndrome is defined by a partial haploid deletion of the long arm of chromosome 5 (del(5q)) in which *RBM22*—along with about 40 genes on average—is lost, making it haploinsufficient. This malignancy is characterized by erythroid hypoplasia and normal to elevated platelet and megakaryocyte counts with hypolobulated nuclei [183]. This is interestingly related to the aforementioned studies of Ebert and coworkers—in which *RBM22* is one of the genes affecting erythroid differentiation the most when experimentally depleted [103]—and Yamauchi and coworkers—in which *RBM22* was identified as essential for the survival of mouse Acute Myeloid Leukemia cell lines [174]. In another study, the haploinsufficiency of *Rbm22* caused by the del(5q) was shown to play a role in the alteration of the hematopoietic differentiation into mouse B cells [184]. Finally, *RBM22* was identified as a target gene of WDR5, which is overexpressed in some Acute Lymphoblastic Leukemia and Acute Myeloblastic Leukemia. The expression of *RBM22* is positively correlated to that of *WDR5*, making it also overexpressed in those cases [185]. Thus, *RBM22* seems to be a major gene of hematopoiesis.

Altogether, these data suggest that even though *RBM22* does not appear to be a driver gene in cancers and other diseases, it seems that it plays an important role in survival, mitosis and differentiation processes, and participates in pathological states. The RNA-splicing and gene expression dependent on RBM22 should therefore be more extensively studied to determine to what extent *RBM22* contributes to oncogenesis or to the phenotype of multigenic diseases.

## 5. Conclusions

*RBM22* is a gene encoding an RNA-binding protein that was discovered two decades ago. Being a fusion of two yeast genes—*Cwc2* and *Ecm2*–, the human *RBM22* encodes a protein containing a Zinc Finger like domain, a Zinc Finger domain, and an RNA Recognition Motif. It was at first described to be involved in pre-mRNA splicing, in which it plays a major, non-redundant role, making it a key protein of the spliceosome. Thus, when it is depleted, the pre-mRNA splicing process is altered, mainly leading to intron retention and exon skipping. Moreover, *RBM22* has recently been described—conjointly with dozens of other RBPs—as a transcription-factor-like protein, being able to bind chromatin in a nascent-RNA-dependent fashion and to regulate the expression of thousands of genes. This dual RNA- and DNA-binding activity gives *RBM22* an essential role in the cell. There are still many questions that need to be addressed about BRM22. Among them, the question of its specific target genes and main target pre-mRNAs in different cellular context (lineages, during cell cycle progression, under stress conditions…) could be investigated by ChIP-seq, RNA immunoprecipitation, RNA ChIP-IT, or equivalent techniques. The identification of the RNA or DNA consensus motifs to be recognized by RBM22, possibly investigated by minigenes or high-throughput techniques, again in different cellular contexts, will be of interest for basic research but also to address the degree of pathogenicity of some SNPs if they happen to affect those motifs. Indeed, being deregulated in several diseases, *RBM22* was repeatedly described to play an oncogenic cooperation role with implications in cell proliferation, migration, and tumor aggressiveness. Understanding those close cooperations in which RBM22 is involved would elucidate comprehension for some diseases progress. Future investigations of RBM22 will undoubtedly provide new insights in the coupling of gene transcription and RNA processing, as well as a better understanding of pathological situations, notably in cancer.

## Figures and Tables

**Table 1:** The members of the RBM gene family are listed in the first column and the family numbers of their RRM(s) are indicated in the corresponding row. The colors correspond to clusters of families based on structural homology.

	IDs of RRM Families						
RBMYA1	0						
RBMX	0						
RBM7	72						
RBM8	60						
RBM11	72						
RBM18	129						
RBM23	32	88					
RBM24	25						
RBM25	21						
RBM28	36	92	112				
RBM34	41	98					
RBM38	25						
RBM39	32	88	52				
RBM44	210						
RBM45	414	149	235				
RBM19	19	29	43	120	2	37	58
RBM12	161	185	2	205			
RBMS	10	133					
RBM17	50						
RBM4	102						
RBM4B	102						
RBM14	102						
RBM15	136	153	243				
RBM16	23						
RBM22	77						
RBM26	87	172					
RBM27	87	172					
RBM30	102						
RBM46	20	82					
RBM47	20	82					
RBM5	130	213					
RBM6	213						
RBM10	130	213					
RBM40	113	169					
RBM41	113						
RBM9	141						
RBM20	46						
RBM21	232						
RBM36	152						
RBM42	81						
RBM48	151						

**Table 2:** Global functions of all RBM-family members.

	Biological Processes				Biological Functions			
	mRNA Splicing	RNA Stability	RNA Transport	Translation	Apoptosis	Cell Cycle	Differentiation	Other Functions
RBMY1A1	[31]						Sperm cells [37]	Sperm motility [38,39]
RBMX	[40]				[41]			Zebrafish brain development [42] Cohesion of sister chromatids [43,44] Genome maintenance [45]
RBM3				[46,47]	[41,48]			DNA Damage Response [49] Circadian cycle [50] Cell proliferation [51,52]
RBM4	[34,53,54]				[53]		Muscle [55] Neuronal [56] Pancreas [57]	Circadian cycle [58]
RBM4B								Circadian cycle [58]
RBM5	[34,59]				[8]	[60]	Sperm cells [61]	Cell proliferation [59]
RBM6	[34,59]				[8]			Cell proliferation [59]
RBM7		[62]						
RBM8A	[63]	[63]	[64]	[65]	[66,67]	[66,67]	Neural [68]	Cell proliferation [68] DNA damage signaling & response [67,69] Cortical development [63]
RBM9	[33]						Pluripotent stem cells [70]	
RBM10	[34,59,71]				[8,72]	[73]		Cell proliferation [59,74]
RBM11	[75]						Neuro/Germinal [75]	
RBM12								
RBM13								
RBM14	[76]							Mouse embryo development [77] DNA damage response [76,78]
RBM15	[79]		[80]		[81]		Blood cells [82,83]	RNA-methylation regulation [84]
RBM16								Transcription [85]
RBM17	[86]				[87,88]			Cell proliferation [88,89]
RBM18								
RBM19							Digestive tube [90,91]	rRNA processing [92] Pre-implantation development of mouse embryo [93]
RBM20	[94]							
RBM21	[95]							RNA poly-adenylation [96]
RBM22	[29,97,98,99,100,101]					[102]	Erythroid [103]	Transcription regulation [104,105] Zebrafish development [90] Drosophila development [106]
RBM23	[107]							
RBM24	[108,109]	[110]		[111]			Embryo [108] Heart [111]	Cell proliferation [112]
RBM25	[113,114,115]				[115]			Cell proliferation [115]
RBM26		[35]						
RBM27		[35]						
RBM28	[116]							
RBM29								Cell hair development in cochlea [117,118]
RBM30								Circadian cycle [58]
RBM33								
RBM34								
RBM36	[119]							
RBM38	[109,120]	[121,122]		[121,123]		[121]	Heart [124] Erythrocytes [120,125]	Cell proliferation [126]
RBM39	[127]							
RBM40	[119]							
RBM41								
RBM42	[128]	[129]				[128]		
RBM43		[130]				[130]		Cell proliferation [130]
RBM44								Meiosis [131]
RBM45	[132]						Neural [133]	
RBM46		[134]						Spermatogenesis [135] Mouse embryo development [134]
RBM47		[136,137]						RNA editing [138] Cell proliferation [136]
RBM48	[139]							

**Table 3:** RBM22 protein partners in the spliceosome.

From Complex	Protein Partner	References
NTC	Cdc5	Zhang et al., 2017 [29]
	Isy1	McGrail et al., 2009 [142]; Hogg et al., 2014 [150]
	PRP19	McGrail et al., 2009 [142]
NTR	Aquarius	Bertram et al., 2017 [153]; Kastner et al., 2019 [155]
	G10	Bertram et al., 2017 [153]; Zhang et al., 2017 [29]
	PRP17	Zhang et al., 2017 [29]
	SKIP	Bertram et al., 2017 [153]; Zhang et al., 2017 [29]
Other	PPIL1	Bertram et al., 2017 [153]; Zhang et al., 2017 [29]; Zhan et al., 2018 [98]
	PRP8	Bertram et al., 2017 [153]
	SLU7	Xu et al., 2001 [161]
